# Nitric oxide and thyroid carcinoma: A review

**DOI:** 10.3389/fendo.2022.1050656

**Published:** 2023-01-09

**Authors:** Yu Huang, Rinkiko Suguro, Wei Hu, Jiayu Zheng, Yawen Liu, Mingxin Guan, Na Zhou, Xin Zhang

**Affiliations:** ^1^ School of Pharmacy, Macau University of Science and Technology, Macau, Macau SAR, China; ^2^ State Key Laboratory of Quality Research in Chinese Medicine, Macau, Macau SAR, China; ^3^ Department of Epidemiology and Biostatistics, School of Public Health, Jilin University, Changchun, China; ^4^ College of Pharmacy, Dalian Medical University, Dalian, China

**Keywords:** nitric oxide, thyroid carcinoma, endocrine system, eitric oxide synthase, treatment

## Abstract

Thyroid carcinoma is the most common endocrine cancer in the world, and its incidence has been steadily increasing in recent years. Despite its relatively good prognosis, therapies have not improved greatly in recent years. Therefore, exploring new therapies for thyroid carcinoma represents an unmet need. Nitric oxide (NO) is a short-term endogenous signaling molecule that plays a vital role in various physiological and pathological processes and is synthesized by nitric oxide synthase (NOS). Many studies have been conducted over the past decades to explain its correlation to cancer. NO exerts a wide range of effects on cancer, involving angiogenesis, apoptosis, cell cycle, invasion, and metastasis. It also serves a dual function by promoting and halting tumor development simultaneously. The relationship between NO and thyroid carcinoma has been intensively studied and discussed. This paper reviews the role and molecular mechanism of NO in thyroid carcinoma and discusses potentials of prevention and treatment of thyroid carcinoma.

## 1 Introduction

The thyroid tumor is the most common endocrine tumor. According to a population-wide epidemiological study of thyroid carcinoma, there are about 449,000 new cases of thyroid carcinoma in women and 137,000 in men in 2020. The corresponding age-standardized incidence is about 10.1 cases per 100,000 women and 3.1 cases per 100,000 men (1).There are many types of thyroid tumors. Classified by the cellular origin, they can be divided into two major categories. One of them is medullary thyroid carcinoma (MTC) caused by C cells (approximately 3-5%), another type of thyroid carcinoma is derived from follicular cells (about 95%) such as papillary thyroid cancer (PTC), follicular thyroid cancer (FTC), poorly differentiated thyroid cancer (PDTC) and aplastic thyroid cancer (ATC). PTC and FTC are considered to be well-differentiated thyroid carcinomas (2, 3). The prognosis of most thyroid carcinomas is reasonably good, and the 10-year survival rate among patients after surgery and other treatments is above 90% (4). Despite this, some other types of thyroid carcinomas are with a high degree of malignancy, and the survival rate is therefore reduced. For instance, the five-year survival rate of PDTC is 66% (5) and the ten-year survival rate of MTC is 62% (6).

The conventional treatment of thyroid carcinoma is usually surgery, which is divided into total thyroidectomy and unilateral thyroidectomy. This is the preferred treatment for most differentiated thyroid carcinomas (7). The choice of surgery, however, remains controversial. People who support total thyroidectomy believe that most patients with thyroid carcinoma have tumor tissue in both lobes of the thyroid gland (8), and thyroid recurrence occurs in the contralateral side in 5%-10% of patients (9). In contrast, proponents of unilateral thyroidectomy argue that more extensive surgery does not improve survival and unilateral surgery is less halmful to the patient than bilateral surgery.

Due to the great differences in the treatment and prognosis of different types of thyroid cancer, it has become an important topic to find a convenient and effective new treatment method.

In the last century, NO was known to be a toxic molecule. However, since the beginning of this century, NO has been increasingly recognized as a signaling molecule involved in a wide range of physiological activities in the human body, such as inflammation, immunity, hormone secretion, platelet aggregation, bronchiectasis, and so on (10, 11). Endogenous NO generation is regulated by nitric oxide synthases (NOS) (12). NO is closely associated with tumors and is often considered to have dual effects on cancer cells, that is, both promoting and inhibiting the growth of cancer cells (13). This review goes over the literature on thyroid carcinoma and NO to find a potential link between the two and to propose the basis for possible new treatment of thyroid carcinoma.

## 2 Physiological function of nitric oxide

Nitric oxide (NO) is an extremely short-lived diatomic free radical molecule with a half-life of less than 5 seconds *in vivo* (14). Nevertheless, NO has a wide range of effects on the human body. Ferid Murad was awarded the Nobel Prize in Physiology or Medicine in 1998 for his finding on cyclic guanosine monophosphate (GMP) dependence.In his early findings, NO was shown to activate the cytoplasmic isoform of guanylyl cyclase, which catalyzes the conversion of guanosine triphosphate (GTP) to cyclic guanosine monophosphate (GMP). In these circumstances, the increase of GMP level will lead to a series of phosphorylation processes, thereby causing the dilation of blood vessels and the change of blood flow velocity (15). This pathway is known as the GMP-dependent pathway. In addition to this, another pathway for NO is the GMP-independent pathway. It produces effects through S-nitrosylation of proteins, the processing which is mainly related to DNA damage and p53 mutation (16).

The effect of NO itself can be divided into direct and indirect effects in most cases. Direct effect means that NO binds to metal complexes of different proteins and interacts with each other to regulate physiological activities (17). The indirect effect refers to the reaction of NO with O_2_ and other endogenous free radicals and the production of reactive nitrogen. They are responsible for participating in other signaling and mediating toxic reactions (18).

Apart from that, NO also has a close relationship with immunity. It is widely considered to be participating in immune activities, regulating the functional activities, growth and death of macrophages, T lymphocytes, antigen-presenting cells, mast cells, neutrophils, NK cells, and other cells (19). NO plays a crucial role in allergy and autoimmune diseases by influencing immunity, especially T cell-mediated immune activity (20). Therefore, for diseases related to the immune system, NO may become a new therapeutic target.

## 3 Nitric oxide and nitric oxide synthase

Nitric oxide synthase (NOS) is a key enzyme in the production of NO in the human body. There are three main subtypes of NOS in the human body, namely nNOS, eNOS, and iNOS. Among them, nNOS is mainly found in the nervous system and is an essential enzyme for neuronal conduction, and eNOS was first discovered in endothelial cells and plays a crucial role in vasodilatation and blood pressure control. Both isomers depend on Ca^2+^ for concentration regulation and produce small amounts of NO for a short period (usually within seconds to minutes). The other type of iNOS is an isomer which is inducible and calcium-independent. It does not persist in cells and is only expressed when cells are stimulated. Unlike the previous two isomers, iNOS can be induced to produce NO continuously. Once induced, it produces large amounts of NO for hours to days (21, 22).

There are many controversies about the physiological role of NOS. Some believe it is a harmful enzyme and is involved in the development of inflammation. Atochina-Vasserman et al. (23), for instance, found that pneumonia in mice was reduced when iNOS was inhibited. Besides this, the findings of Miyoshi et al. (24) have shown that iNOS promotes low-density lipoprotein oxidation through the activation of smooth muscle cells, which is a key step in the formation of atherosclerosis. In the nervous system, NOS activity is high. When the expression of NOS itself is overactivated, large amounts of NO are produced, mediating cytotoxic effects and leading to nerve damage or even death (25).

With the in-depth study of NOS, more new findings have shown that NOS also brings many beneficial effects. nNOS is widely found in neurons, astrocytes, and cerebrovascular, and is believed to be involved in the regulation of learning, memory, neural development, and other physiological activities (26), though many previous studies have shown that NO produced by nNOS in the brain harms neural development, especially on neurons (27). Study by Lou et al. (28) have shown that when nNOS inhibitors are administered to neural stem cells (NSC), their ability to differentiate into neurons and astrocytes is inhibited. Weitzdoerfer et al. (29) found cognitive performance impairment in nNOS knockout mice, which suggests that nNOS may affect NSC differentiation. These findings indicate that both NOS and NO play a wide range of important roles in human physiological activities.

## 4 Nitric oxide and cancer

**Figure 1:**
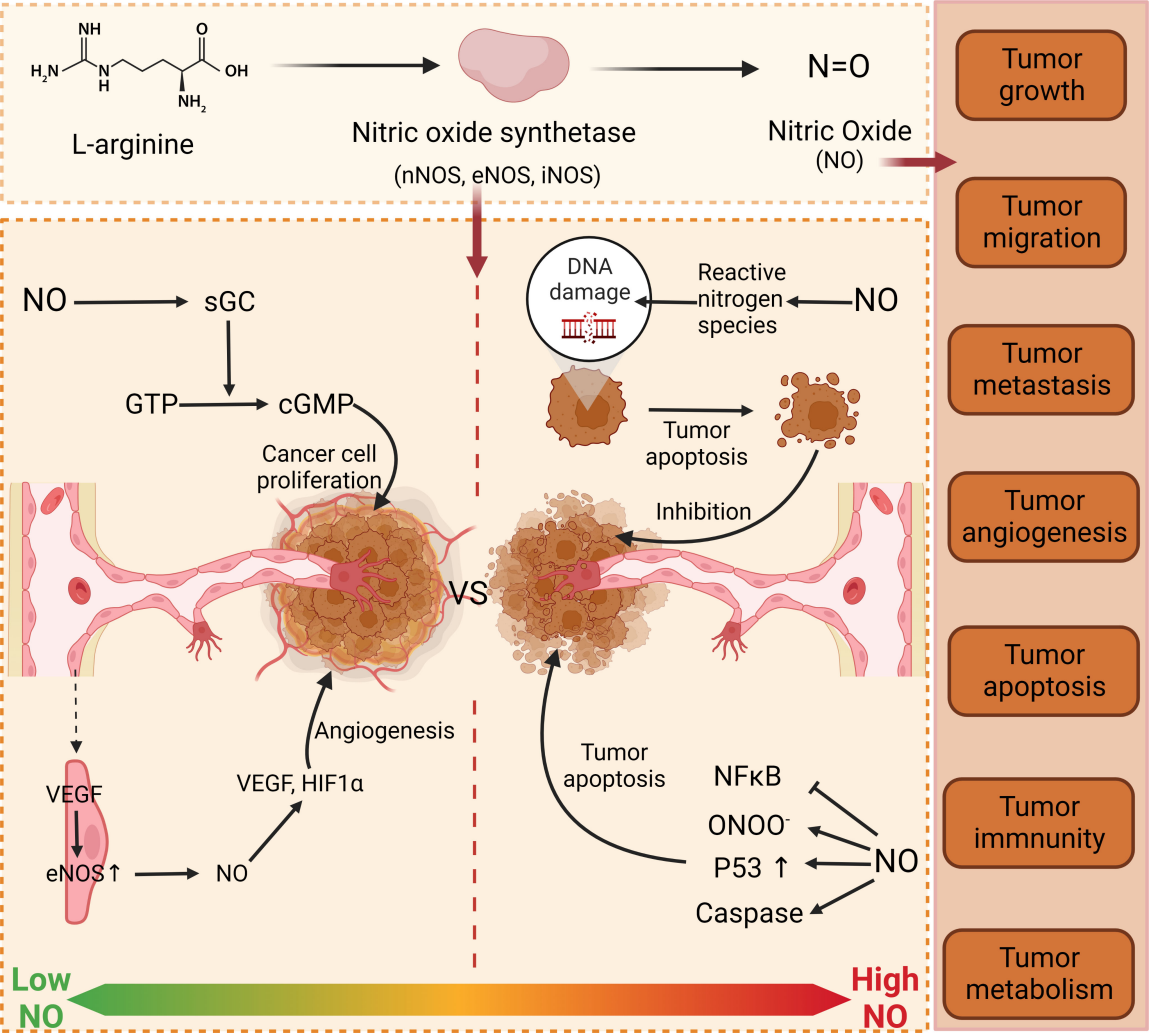
Function of NO in cancer. NO has dual properties on tumor cells. At low concentration, NO promotes tumor growth and proliferation through GMP-dependent pathway and angiogenesis. At high concentration, NO destroys the DNA of tumor cells by releasing active nitrogen, thus achieving the purpose of inhibiting tumor cells. Furthermore, NO can also accumulate P53 to affect the physiological processes of tumor cells. nNOS, neuronal nitric oxide synthase; eNOS, Endothelial Nitric Oxide Synthase; iNOS, Inducible Nitric Oxide Synthase; NO, nitrous oxide; sGC, Soluble guanylate cyclase; GTP, Guanosine Triphosphate; cGMP, cyclic guanosine monophosphate; VS, versus; VEGF, Vascular Endothelial Growth Factor; HIF1A, Hypoxia inducible factor-1α; DNA, deoxyribonucleic acid; NFkB, nuclear factor κB; ONOO, peroxynitrite anion.

For a long time, a large number of researchers have been exploring the relationship between NO and cancer. Numerous studies have shown that the effects of NO on cancer cells are bidirectional. At low concentrations, it can protect cancer cells and accelerate tumor development. When the concentration of NO exceeds the ideal concentration for tumor growth, it results in growth arrest or apoptosis (30). In some *in vitro* experiments, it was found that the cell differentiation rate increased and the cell differentiation decreased at low doses of NO. However, at higher concentrations of NO, cell growth was shown to decrease, while cell differentiation was shown to increase (31). These findings also demonstrate that the effect of NO on tumors is bidirectional. NO can regulate all aspects of tumor physiological activities, including tumor growth, migration, invasion, survival, angiogenesis, and metastasis (32). These processes are usually determined by many factors, such as the concentration of NO, the time when tissue cells are exposed to NO, and the environment of redox reactions (30, 33). At low concentrations, it can stimulate the growth and proliferation of tumor cells (34) to achieve the purpose of promoting tumor growth. Apart from that, NO can also promote angiogenesis. It is well known that angiogenesis is the generation of new blood vessels from original blood vessels, which is an important step in tumor progression and metastasis (35). NO promotes angiogenesis, thereby helping tumor growth and metastasis (36). However, this mechanism is quite complicated and consists of the following processes. eNOS stimulates endothelial expression to produce and release NO. With the help of vascular endothelial growth factor (VEGF), eNOS expression is up-regulated, thereby releasing more NO and promoting the differentiation of quiescent endothelial cells into vessels (**Figure 1**) (37), to promote angiogenesis. At high concentrations, NO can mediate apoptosis. As a transcription factor, P53 is an important signaling molecule in cells, which regulates cell growth, apoptosis, DNA repair, and other processes by changing gene expression, and it is regulated by NO as well (38, 39). NO can lead to the accumulation of wild-type P53, thus achieving the purpose of inhibiting the growth of tumor cells (40). Interestingly, there is a negative feedback regulation between P53 and iNOS, in which P53 controls NOS2 activities. This relationship exists in many cancers (41, 42). These studies have proved that NO is a key factor in the physiological activities of tumor cells. It can be adjusted to control its flux in tumor tissues to achieve the purpose of tumor treatment, and it can achieve the purpose of tumor treatment by adjusting and controlling its flux in tumor tissues. In recent studies, NO has been used in cancer therapy alone or in combination, and has been shown to have immune-boosting, photodynamic therapeutic effects (43–45). Jiang et al. (46) found that NO is an immunogenic cell death (ICD) inducer, which can trigger a strong specific antitumor response, thereby enhancing the effect of tumor immunotherapy. Photodynamic therapy (PDT) is based on photosensitizers (PS) that transfer energy to tissue oxygen under specific laser irradiation to generate cytotoxic reactive oxygen species (ROS) that selectively kill tumor cells. The antioxidant glutathione (GSH) in tumor cells can effectively remove ROS and thus affect the effect of PDT. In addition, the study of Yao et al. (47) showed that NO could consume GSH, alleviate cellular hypoxia and improve the efficacy of PDT. The matter of the delivery mode of NO was examined in detail in the paper written by Alimoradi et al. (48).

## 5 Nitric oxide and thyroid carcinoma

The relationship between NO and thyroid is very intimate. NO is thought to be involved in many thyroid diseases, such as hypothyroidism and hyperthyroidism. In hypothyroidism, researchers have found that NO plays a quite important role in the damage of different tissues (49, 50). During the early stage of fetal development, maternal thyroid hormone (TH) is the crucial element to maintain normal brain and thyroid development (51, 52). In addition, nNOS is a key target gene of maternal TH in fetal cerebral cortex development. Maternal hypothyroidism may lead to fetal neural hypoplasia (53), while hyperthyroidism may affect cardiac function (54). Rodriguez et al. found that NO may modulate the response to hyperthyroidism, leading to cardiovascular dysfunction, even though this may be related to gender (55). The above studies proved that NO could regulate a large number of physiological processes of the thyroid and had a significant influence on it.

Thyroid carcinoma, as an endocrine tumor, is a malignant thyroid lesion. Kayser et al. detected the expression of NOS, iNOS and eNOS in different tissues, and found that the expression of NOS was much higher in thyroid tumor tissues (56). Choe et al. (57) found that iNOS expression was low in normal follicular epithelial cells, but was significantly enhanced in all follicular epithelial derived thyroid tumors. Maria et al. found that NOS enzyme can be expressed in WRO cells, which may be involved in the growth and progression of thyroid tumors (58). These studies indicate that there may be a clinical correlation between NO and thyroid tumors, but the specific molecular mechanism has not been elucidated. However, a study performed by Patel et al. (59) about the relationship between thyroid tumors and NOS in adolescents and children has shown that the contents of iNOS and eNOS in diseased thyroid tissues in PTC and FTC were higher than those in surrounding normal thyroid tissues, and this result was statistically significant. Additionally, they also found that VEGF expression was upregulated in tumor tissue. The study of Nakamura et al. (60) also confirmed that VEGF-D expression may be regulated by NO. This is consistent with the previously described evidence that NOS promotes angiogenesis to help tumor growth. As a consequence, it is demonstrated that NOS/NO plays a crucial part in thyroid tumors.

**Figure 2:**
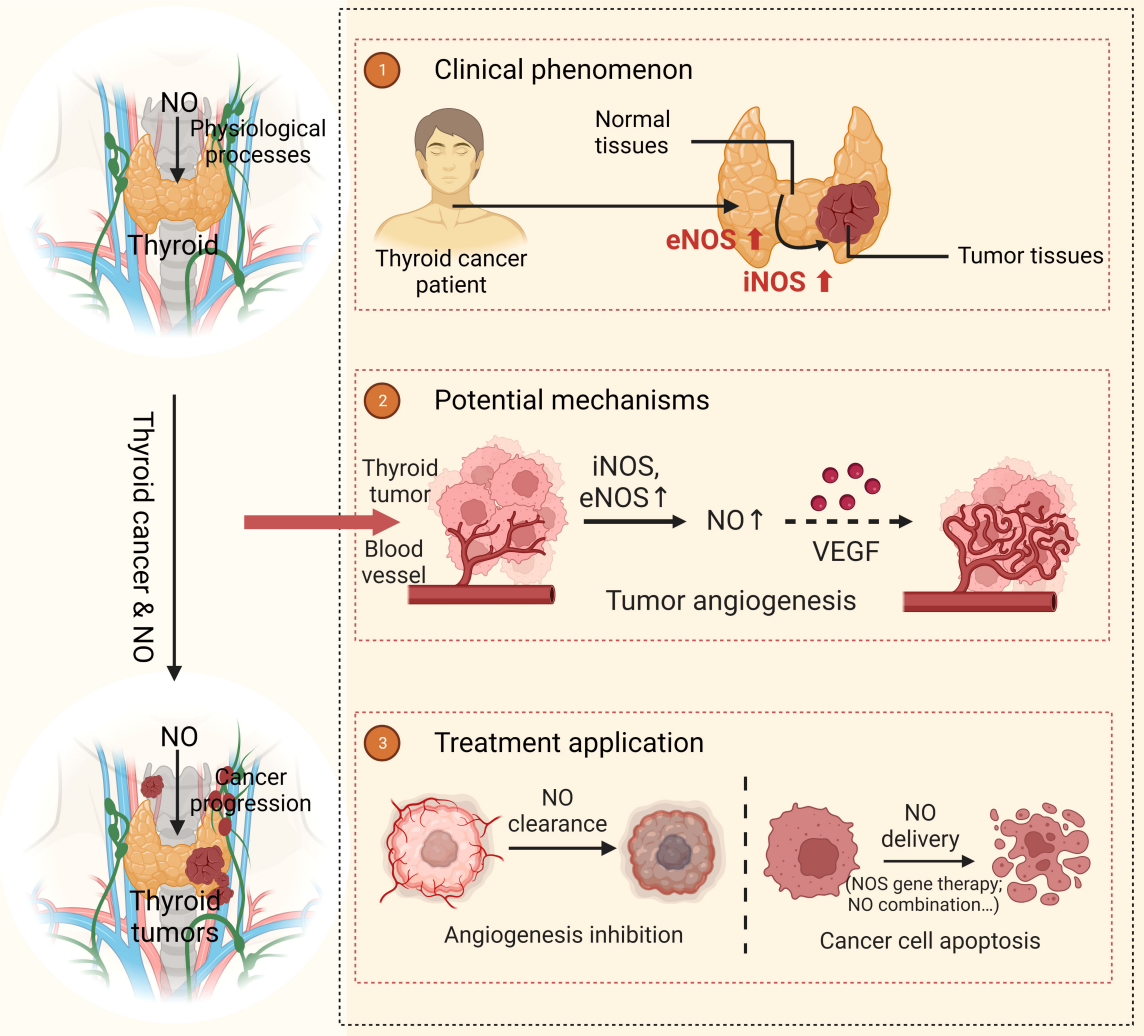
Function of NO in thyroid carcinoma The expressions of eNOS and iNOS in thyroid carcinoma tissues are higher than those in normal thyroid tissues. As a result, this increases the content of NO, which promotes tumor growth by regulating angiogenesis and VEGE. When NO is targeted to the thyroid tissue and maintained in high concentration, it can inhibit the growth and proliferation of thyroid tumors and therefore achieve therapeutic goals. NO, nitrous oxide; NOS, Nitric Oxide Synthase; eNOS, Endothelial Nitric Oxide Synthase; iNOS, Inducible Nitric Oxide Synthase; VEGF, Vascular Endothelial Growth Factor.

Since NO has a wide range of effects in thyroid tumors, regulation of NOS/NO may become a new convenient and economic treatment for thyroid tumors. Studies have shown that NO produced by iNOS has a great lethal effect on MTC cells and the volume of tumor tissues is significantly reduced (35%, P <0.05) by direct plasmid insertion of DNA-NOS II gene into MTC mice (61). The above experiments indicate that iNOS gene seems to be a promising gene therapy for human cancer. In another study, two nonsteroidal anti-inflammatory drugs (celecoxib and indomethacin) were added to the supply of NO to inhibit the growth of MTC cells. The results showed that compared with using a single drug, the therapeutic effect of any combination drug therapy was better than that of a single NO donor (62). The above studies indicated that the regulation of NOS/NO could play a role in the treatment of thyroid carcinoma. Beyond that, a new study in 2022 conducted by Sun et al. (63) explored the antitumor mechanism of nano-pulse stimulation (NPS) on human ATC cells *in vitro*. *in vitro* In this study, it was found that NPS could participate in the killing of ATC cells by iNOS. NPS can increase the activity of iNOS through the structure of enzyme, which greatly increases the concentration of NO. High levels of NO block the electron transport chain in mitochondria, leading to mitochondrial apoptosis and the release of cytochrome C, which activates the caspase process and guides cancer cell apoptosis. These findings also have a great reference value for researchers to develop new, convenient, effective, and cost-effective treatment regimens and therapeutic drugs (**Figure 2**).

## 6 Conclusions

This paper reviews the literature on NO and thyroid carcinoma and explains the main association between the two. NO is a signaling molecule widely involved in human physiological activities. It is produced and released by the NOS enzyme family, and the effect is significantly bidirectional (64). It participates in every physiological process of various tumors, especially in the angiogenesis of tumor tissues, which has also been proved in thyroid tumors. At present, the mainstream treatments for thyroid tumors are surgery and the standard method of postoperative radioiodine (65). However, surgery often brings physical pain and psychological fear to patients in clinical practices. Beyond that, the need of receiving radioiodine therapy after surgery may lead to even more psychological stress on patients. Therefore, it is an unmet need to find a convenient, effective, and therapeutic potential strategy. on this ground t he therapeutic potential of NOS/NO in tumors is obvious. NO has been used in the treatment of various cancers, such as lung cancer, breast cancer, melanoma, and so on (66). If NO is to be considered as a therapeutic agent for thyroid tumors, two aspects should be considered due to its inherent biphasic nature. One aspect is to keep thyroid tumor tissue below the optimal NO concentration for growth. High expression of NOS is detected in diseased thyroid tissues, which also produce higher concentrations of NO than normal thyroid tissues. It is possible that NO at this concentration may stimulate cell proliferation and angiogenesis to promote the growth and development of tumor cells. Another aspect is to keep a high concentration of NO in thyroid tumor tissue to achieve the concentration of promoting cell apoptosis, to achieve the inhibitory effect on tumor cells. The animal experiments mentioned above also prove that this method is feasible. With the in-depth research on NO, researchers have realized its importance for tumor treatment, and more and more NO delivery systems have been developed (48). This greatly improves the target organ delivery in the treatment of different tissues and different types of tumors, making it more accurate. Meanwhile, it also reduces the effect of NO on other tissues and organs, improving the therapeutic effect. However, there have only been a few experiments on NO, and NO’s exact effects are still unknown. More experiments, especially *in vivo*, are needed to verify the real effects of the treatment in the future. On all accounts, the approach of NO pathway regulation should be recognized. Compared with conventional surgical radiotherapy and chemotherapy, NO pathway regulation has fewer side effects, and patients will suffer less mental and physical stress from it. As a consequence, the treatment of thyroid carcinoma with NO alone or in combination may be a potential better treatment method to improve the therapeutic effect of patients and improve their quality of life.

## Author contributions

YL, XZ, NZ designed the structure of the article and collected the main reference; YH, MG and RS analyzed the data. JZ, YH, and XZ wrote the manuscript, WH substantially revised the manuscript. All authors contributed to the article and approved the submitted version.
